# Transcriptional Response to Standard AML Drugs Identifies Synergistic Combinations

**DOI:** 10.3390/ijms241612926

**Published:** 2023-08-18

**Authors:** Piyush More, Joëlle Aurelie Mekontso Ngaffo, Ute Goedtel-Armbrust, Patricia S. Hähnel, Udo F. Hartwig, Thomas Kindler, Leszek Wojnowski

**Affiliations:** 1 Department of Pharmacology, University Medical Center, Johannes Gutenberg-University, 55131 Mainz, Germany; joelle.mekontso@leibniz-inm.de (J.A.M.N.); goedtela@uni-mainz.de (U.G.-A.); wojnowsk@uni-mainz.de (L.W.); 2 Leibniz Institute for New Materials, 66123 Saarbrücken, Germany; 3 University Cancer Center (UCT) Mainz, Johannes Gutenberg-University, 55131 Mainz, Germany; p.haehnel@uni-mainz.de (P.S.H.); thomas.kindler@unimedizin-mainz.de (T.K.); 4 Department of Hematology & Medical Oncology, University Medical Center, Johannes Gutenberg-University, 55131 Mainz, Germany; uhartwig@uni-mainz.de; 5 Research Center of Immunotherapy, University Medical Center, Johannes Gutenberg-University, 55131 Mainz, Germany

**Keywords:** AML, transcription, gene expression changes, cytarabine, daunorubicin, combination therapy

## Abstract

Unlike genomic alterations, gene expression profiles have not been widely used to refine cancer therapies. We analyzed transcriptional changes in acute myeloid leukemia (AML) cell lines in response to standard first-line AML drugs cytarabine and daunorubicin by means of RNA sequencing. Those changes were highly cell- and treatment-specific. By comparing the changes unique to treatment-sensitive and treatment-resistant AML cells, we enriched for treatment-relevant genes. Those genes were associated with drug response-specific pathways, including calcium ion-dependent exocytosis and chromatin remodeling. Pharmacological mimicking of those changes using EGFR and MEK inhibitors enhanced the response to daunorubicin with minimum standalone cytotoxicity. The synergistic response was observed even in the cell lines beyond those used for the discovery, including a primary AML sample. Additionally, publicly available cytotoxicity data confirmed the synergistic effect of EGFR inhibitors in combination with daunorubicin in all 60 investigated cancer cell lines. In conclusion, we demonstrate the utility of treatment-evoked gene expression changes to formulate rational drug combinations. This approach could improve the standard AML therapy, especially in older patients.

## 1. Introduction

Drug combinations provide an effective way to tackle the resistance of cancer cells to individual drugs and to reduce side effects by means of dose reduction. Taking acute myeloid leukemia (AML) as an example, the combination of cytarabine and daunorubicin forms the first-line induction therapy that achieves complete remission in 80% of the patients [1]. But most older patients, which form most AML cases, are unfit to receive this standard therapy due to toxicities associated with high-dose cytarabine [2]. Second-line, less toxic therapies consisting of hypomethylating agents, such as azacitidine, has been approved for such cases [3]. Another limitation of the standard induction therapy is that up to 35% of patients with favorable risk profiles and 80% of patients with unfavorable risk profiles eventually relapse, limiting the use of the standard drug combination due to acquired resistance [4,5]. Hence, identifying safer and optimal drug combinations is a major need to improve AML treatment outcomes, especially in older patients.

The typical approach to identifying drug combinations includes high-throughput in vitro and/or ex vivo drug cytotoxicity screening. However, the experimental evaluation of hundreds of drugs and their combinations is laborious. Hence novel predictive methods are required to shortlist the most promising candidates that can be verified experimentally. Gene expression profiles have been underutilized in clinical practice to formulate drug combinations compared to somatic mutations. For example, the standard AML therapy is supplemented in carriers of FLT3, IDH1/2, and BCL2 mutations by targeted drugs [6]. However, the use of gene expression profiles has been limited in defining the molecular identity of cells. Recently, pre-treatment gene expression profiles have been demonstrated to improve AML classification [7]. These transcriptomics-derived sub-groups were even associated with distinct prognoses and drug sensitivities. Gene expression profiles have also been explored to identify specific biomarkers defining AML aggressiveness, the likelihood of relapse, and to predict the treatment response [8,9,10].

However, such gene expression-based markers are still far away from clinical implementation. Crucially, their utility in treatment refinements has not been explored, although it is known that the transcriptional effects contribute to the drug’s anticancer activity [11]. The most likely reason for the lack of clinical translation is that treatment-relevant gene expression profiles become apparent only after the treatment onset. Considering that chemotherapeutic drugs typically inhibit cancer cells by altering multiple molecular processes, capturing their effect on global transcription possesses immense potential in drug repurposing [12]. Thus, we have shown that the transcriptional changes in response to topoisomerase II poison etoposide enhance its cytotoxic effects mediated via primary DNA double-strand breaks. We further utilized these transcriptional changes to identify etoposide combination partners [13].

In the present work, we analyzed the transcriptional response of AML cells to first-line drugs cytarabine and daunorubicin as monotherapies and in combination by performing RNA sequencing. By comparative analysis, we identified response-defining gene expression signatures that were mimicked pharmacologically to identify potential novel daunorubicin-based combinations.

## 2. Results

### 2.1. AML Cell Lines Exhibit Variable Response to Standard Drugs

**Figure 1:**
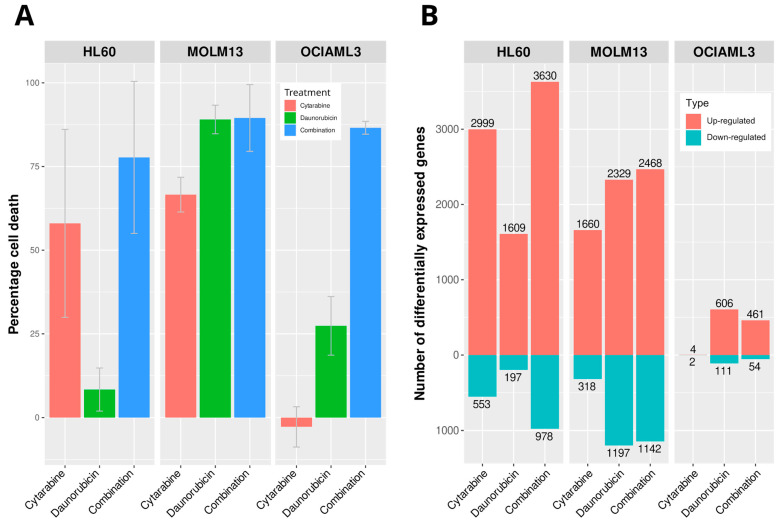
Response of AML cell lines to standard drugs. (**A**) Percentage cell death and (**B**) number of gene expression change in 3 AML cell lines in response to treatments with cytarabine, daunorubicin, and their combination for 24 h compared to DMSO. Percentage cell death was measured using CellTiter-Glo assay, and gene expression changes were identified from RNA-Seq.

We treated HL60, MOLM13, and OCIAML3 cells with cytarabine (0.1 µM), daunorubicin (0.02 µM), and their combination (0.1 µM cytarabine + 0.02 µM daunorubicin) for 24 h. CellTiter-Glo-based cell cytotoxicity measurements revealed differential sensitivity of these cells to individual drug treatments. HL60 cells exhibited the lowest response to daunorubicin treatment (percentage cell death 7 ± 7.02), while OCIAML3 did not respond to cytarabine treatment (Figure 1A). On the other hand, MOLM13 cells responded to both cytarabine (percentage cell death 66.6 ± 5.6) and daunorubicin (percentage cell death 91.8 ± 4.7) treatments. All three cell lines responded similarly to the combination of cytarabine and daunorubicin (percentage cell death 77.7 ± 25.2, 89.5 ± 10.9, and 86.6 ± 1.3 for HL60, MOLM13, and OCIAML3, respectively).

### 2.2. Cytotoxic Response of AML Cells Correlates with the Transcription Response

To understand the transcriptional basis for drug sensitivity and synergy, we quantified gene expression by performing total RNA sequencing after treatment with cytarabine and daunorubicin as monotherapies and after the combination therapy. We compared these profiles with DMSO-treated cells to identify the gene expression changes. Most transcriptional changes in AML cells were gene inductions (average 79%); as opposed to gene repressions (average 21%). This proportion was consistent across both individual treatments and the combination treatment (average percentage of down-regulated genes 21.7, 20.1, and 21.1 and average percentage of up-regulated genes 78.3, 79.9, and 78.9 for cytarabine, daunorubicin, and combination, respectively). The total number of differentially expressed genes correlated with the percentage of cell death in response to drug treatments. HL60 cells responded least to the daunorubicin treatment and exhibited the least number of differentially expressed genes (1806) compared to cytarabine (3552) and the combination (4608). The highest number of differentially expressed genes in response to the combination was associated with the highest cytotoxicity (Figure 1A,B).

Similarly, OCIAML3 cells with no response to cytarabine treatment exhibited only six differentially expressed genes. MOLM13 cells also exhibited an increasing number of differentially expressed genes with increasing cytotoxicity to cytarabine, daunorubicin, and the combination. This indicated that the cytotoxic response was reflected by the transcriptional response of AML cell lines to the treatments.

To further understand the role of gene expression changes in drug-mediated cytotoxicity, we investigated the down-regulation of the genes essential for cell survival. We obtained the list of genes essential for the survival of HL60, OCIAML3, and MOLM13 cells from an RNAi-based cell viability screen DepMap [14,15]. Several essential genes turned out to be down-regulated. In MOLM13 and OCIAML3 cells, daunorubicin down-regulated the highest number of essential genes (510 and 15, respectively; Supplementary Figure S1). In contrast, in daunorubicin-resistant HL60 cells, daunorubicin down-regulated the least number of essential genes (45) compared to 137 by cytarabine and 270 by the combination treatment. Analogously in cytarabine-resistant OCIAML3 cells, no essential gene was down-regulated by cytarabine treatment. These results suggested the involvement of gene expression changes in driving cytotoxic response besides the primary action of the drug.

### 2.3. Transcription Response of AML Cells Vary between Individual Drugs and the Combination

**Figure 2:**
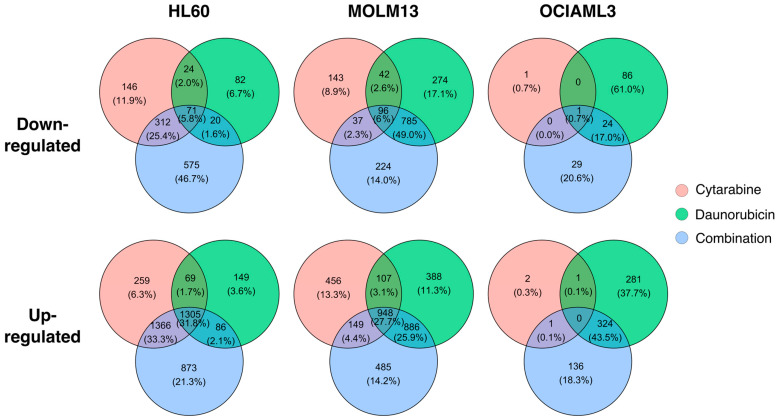
Cell-wise overlapping gene expression changes. The Venn diagrams (unscaled) represent the overlaps among gene expression changes after treatment with cytarabine, daunorubicin, and the combination for 24 h in each AML cell line. In each panel, gene expression changes from all 3 treatments account for 100%.

To investigate the similarities between standard AML drugs and their combination at the transcriptional level, we compared the differentially expressed genes across the three AML cell lines. There were, on average 4% down-regulated and 20% up-regulated genes common to all treatments (Figure 2). In HL60 cells, the transcriptional response to cytarabine and the combination treatment was more comparable (31.2% down- and 65.1% up-regulated genes common) than the response to cytarabine and daunorubicin (7.8% down- and 33.5% up-regulated genes common) and daunorubicin and the combination (7.4% down- and 33.9% up-regulated genes common). In contrast, the transcriptional response of MOLM13 and OCIAML3 cells showed the most overlap between daunorubicin and the combination (55% down- and 53.6% up—regulated genes common in MOLM13 and 17.7% down- and 43.5% up-regulated genes common in OCIAML3) (Figure 2). These were also the cell lines with higher cytotoxic response to daunorubicin than HL60 cells (Figure 1A). On average, the proportion of differentially expressed genes from individual drugs in the combination was 32.8%, 57.3%, and 17.7% for down- and 67.2%, 58%, and 43.6% for up-regulated genes in HL60, MOLM13, and OCIAML3 cells respectively.

The comparison between different AML cell lines revealed few similarities. There were 0%, 0.1%, and 0.1% common down-regulated genes and 0%, 2.9% and 2.8% common up-regulated genes after cytarabine, daunorubicin and the combination treatment, respectively, across 3 AML cell lines (Supplementary Figure S2). Regarding cytarabine response, HL60 and MOLM13 cells were more comparable (9.7% down- and 24% up-regulated genes common) than cytarabine-resistant OCIAML3 cells. A similar trend was observed with daunorubicin and the combination treatments, where HL60 and MOLM13 cells were more comparable than OCIAML3 cells (Supplementary Figure S2). In general, AML cell lines exhibited low overlap among the treatments.

### 2.4. Pathway Annotation of the Gene Expression Changes

Because the transcriptional effect of standard AML drugs and their combination was unique to every cell line, we investigated the associated biological processes to understand further the mechanism of action of individual drugs and the synergy between them. We performed functional enrichment analysis using the clusterProfiler R package to identify biological processes affected by the treatments. The specific pathways were considered to be affected by the treatment if they were affected in all three cell lines by the combination treatment or affected in at least two cell lines by individual treatments. The reasoning behind this approach was that we observed the cytotoxic response to the combination treatment by all cell lines, but at least one cell line was resistant to the individual treatments.

**Figure 3:**
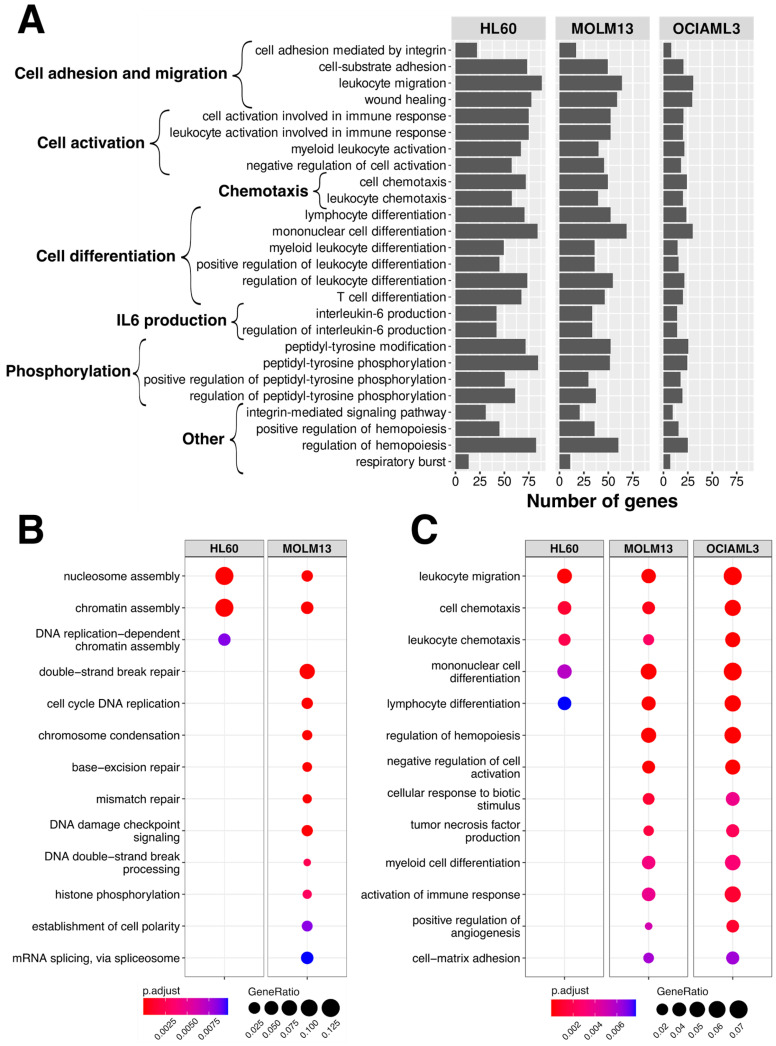
Pathway enrichment analysis. (**A**) Biological processes are commonly affected by all three treatments. The bars represent the number of genes associated with a particular pathway averaged across all three treatments. (**B**) Biological processes related to down-regulated and (**C**) up-regulated genes after daunorubicin treatment for 24 h.

We observed the involvement of processes related to cell adhesion and migration, cell activation, chemotaxis, cell differentiation, IL6 production, and phosphorylation after treatment with both individual treatments and the combination treatment in all 3 AML cell lines (Figure 3A). We further identified pathways uniquely affected by specific treatments. Pathways involved in calcium ion homeostasis, ERK1 and ERK2 signaling, and pyrimidine catabolic processes were activated in response to cytarabine and the combination treatment but not to the daunorubicin treatment (Supplementary Table S1). Daunorubicin and the combination treatment affected IL-6 production. There were certain pathways affected by specific treatments and in specific cell lines. In MOLM13 cells, the genes down-regulated after daunorubicin treatment were enriched for the pathways associated with DNA damage and repair processes, such as double-strand break repair, DNA damage checkpoint signaling, and histone phosphorylation (Figure 3B). These pathways were not associated with daunorubicin-treated HL60 cells. Analogously in MOLM13 and OCIAML3, but not in HL60 cells, genes up-regulated by daunorubicin treatment were involved in cell activation and differentiation processes (Figure 3C). Functional enrichment analysis using g: Profiler revealed the association of several transcriptional factors with the treatment. Notably, only transcription factors AP-4, p53, SMAD4, and THAP1 were associated with daunorubicin-treated OCIAML3 cells, and no transcription factor was associated with the OCIAML3 cells treated with the combination treatment (Supplementary Figures S3 and S4).

**Figure 4:**
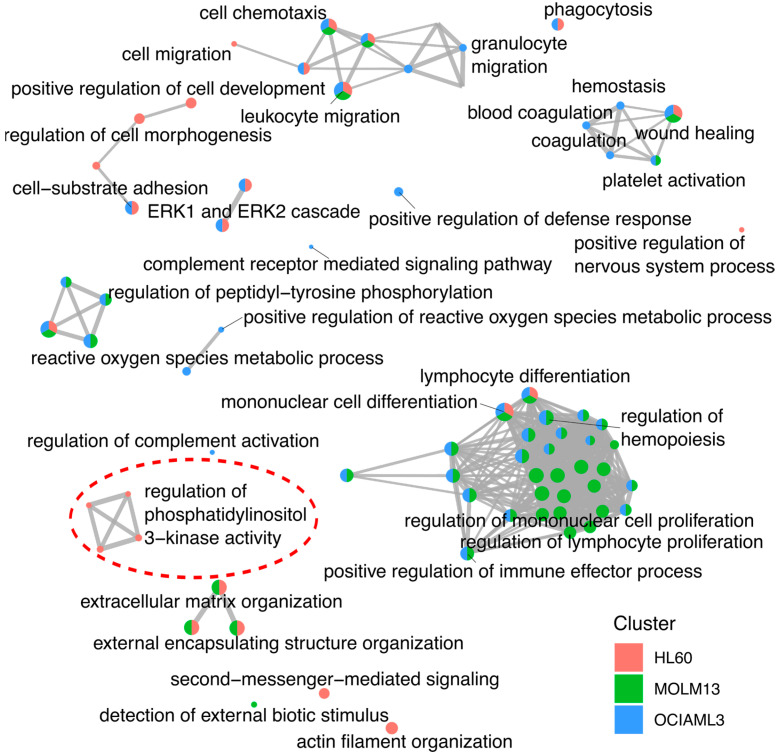
Gene network enrichment map. A network organizing enriched biological processes into a network with edges connecting overlapping gene sets. The network clustered mutually overlapping gene sets together. The clusters represent enriched biological processes after daunorubicin treatment in 3 AML cell lines. The highlighted cluster (dashed red ellipse) indicates the activation of PI3K pathway-related processes in only HL60 cells.

We then formed a network map of daunorubicin-affected biological processes by clustering overlapping gene sets. The clustering showed that the processes related to leukocyte activation and differentiation were predominantly affected by the daunorubicin treatment in MOLM13 and OCIAML3 cells (Figure 4). Interestingly, only HL60 cells exhibited activation of phosphatidylinositol 3-kinase (PI3K) pathway after daunorubicin treatment. PI3K-related pathways were not affected in HL60 by the combination treatment (Supplementary Figure S5).

### 2.5. Gene Expression Changes Predict Daunorubicin Combination Partners

To further understand the lower response of HL60 cells to daunorubicin treatment, we compared its transcriptional response with that of the daunorubicin-sensitive MOLM13 and OCIAML3 cells. We constructed profiles of gene expression changes depicting either response (DNRResponse) or resistance (DNRResist) to daunorubicin treatment. These profiles were constructed assuming the gene expression changes associated with a positive treatment response would have only occurred in daunorubicin-sensitive MOLM13 and OCIAML3 cells. Conversely, the gene expression changes related to a lack of treatment response would have only occurred in daunorubicin-resistant HL60 cells. DNRResist profile consisted of 132 gene inductions and 64 gene repressions, which occurred in HL60 cells after treatment with daunorubicin only and were absent after both daunorubicin and the combination treatment in the other two cell lines (Supplementary Table S2). On the other hand, the DRNResponse profile consisted of 43 gene inductions and two gene repressions observed in OCIAML2 and MOLM13 cells but absent from HL60 cells after both daunorubicin and the combination treatment (Supplementary Table S3). We then analyzed the biological pathways associated with DNRResist and DNRResponse profiles. The genes induced in the DNRResist profile were associated with calcium ion-dependent exocytosis and lipid biosynthetic processes, while gene inductions from the DNRResponse group were associated with nucleosome and chromatin assembly (Supplementary Table S4).

**Figure 5:**
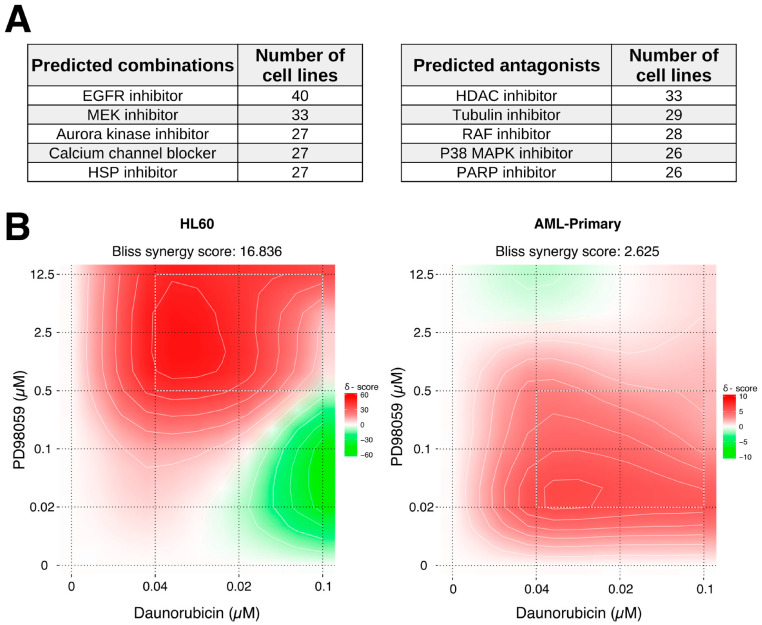

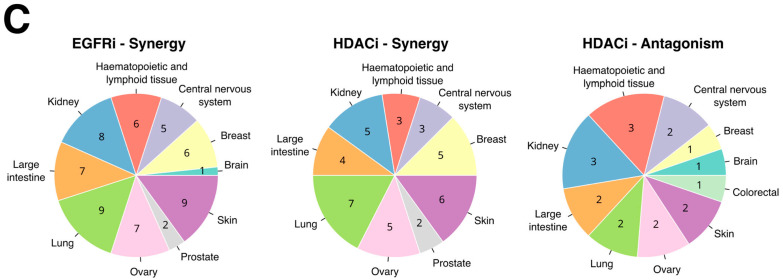
Predicted daunorubicin combinations and their validation. (**A**) Top 5 drug classes predicted to exhibit either synergistic or antagonist response in combination with daunorubicin. Predicted combinations showed a similar transcriptional response to the DNRResponse profile and an opposite transcriptional response to the DNRResist profile. (**B**) Contour plots with the demonstrated effect of combining daunorubicin with MEK inhibitor PD98059 in HL60 and primary AML cells, respectively. (**C**) Pie charts represent either synergistic or antagonist response of 60 cancer cell lines to combining daunorubicin with either EGFR or HDAC inhibitors. Data was obtained from the NCI ALMANAC drug combination screen.

We then hypothesized that the response of HL60 cells to daunorubicin treatment could be enhanced by inhibiting the DNRResist profile and mimicking the DNRResponse profile by adding appropriate drug combinations. To predict the most suitable combination partners, we compared the DNRRes and DNRResponse profiles with the gene expression changes associated with other drug treatments using the CLUE platform. We specifically searched for the drugs with opposite transcriptional responses as that of the DNRRes profile and similar transcriptional responses as that of the DNRResponse profile. We discovered that the transcriptional responses to HDAC, tubulin, RAF, P38 MAPK, and PARP inhibitor were similar to DNRRes, while the response of EGFR, MEK, aurora kinase, and HSP inhibitor and calcium channel blocker was opposite to that of the DNRRes signature (Figure 5A and Supplementary Tables S5 and S6). We predicted these drug classes to be ideal combination partners, assuming the involvement of DNRRes genes in daunorubicin resistance.

### 2.6. Predicted Combinations Exert Synergistic Response in AML Cells

To validate the predicted combinations, we measured the cytotoxic response of HL60, MOLM13, OCIAML3, and primary AML cells to PD98059, a MEK inhibitor, daunorubicin, and their combination. This was compared with the combination of vorinostat, an HDAC inhibitor and a non-ideal candidate combined with daunorubicin (Figure 5A). The CellTiter Glo assay revealed a highly synergistic response to PD98059 and daunorubicin combination (BLISS independence score 16.8), as opposed to the combination of vorinostat and daunorubicin (BLISS independence score 3.3) (Supplementary Figure S6C). Interestingly, PD9809 synergized with daunorubicin with very little standalone cytotoxicity (92% cells viable at the highest tested concentration of 12.5 µM) (Supplementary Figure S7). Furthermore, a primary AML sample exerted a synergistic response (BLISS independence score 2.6) over a wide concentration range of the PD98059 and daunorubicin combination (Figure 5B). MOLM13 cells also exhibited a synergistic response to the combination treatment (BLISS independence score 1.7, Supplementary Figure S6A).

On the contrary, OCIAML3 cells exhibited an antagonist response at a lower concentration of PD98059 (BLISS independence score −1.5, Supplementary Figure S6B). To validate other predicted combination partners in various cancer cell lines, we interrogated the publicly available data of large-scale combination screens. We obtained the data for the combinations of daunorubicin with EGFR and HDAC inhibitors in 60 cancer cell lines. All 60 cancer cell lines exhibited a synergistic response to combining daunorubicin and EGFR inhibitors (Figure 5C and Supplementary Table S7). On the contrary, there were only 29 cancer cell lines with synergistic response and 13 cancer cell lines with antagonist response to the combination of daunorubicin and HDAC inhibitors.

To understand the role of gene mutations in gene expression-based drug combinations, we analyzed their correlation by fitting an ANOVA model. For pan-cancer analysis (using 56 cancer cell lines), there were 59 genetic alterations with significant association with increased daunorubucin-EGFR inhibitor synergy (Supplementary Table S8). Among these, *NUP107*, *PATZ1*, *SBNO2*, *SRRT*, *ZNF324B*, *ZNF595*, and *ZNF646* were associated with the regulation of transcription and *IGF1R*, and *PRKCD* were associated with the cellular response to chemicals (Supplementary Figure S8). We further analyzed the top ten frequently mutated genes in AML (obtained from cBioportal). None of these genes was significantly associated with the extent of daunorubicin-EGFR inhibitor synergy, as evident from the comparable number of cell lines with higher and lower synergy against respective mutations (Supplementary Table S9). Among these, eight cell lines with *DNMT3A* mutations exhibited a higher daunorubicin-EGFR inhibitor synergy than only three cell lines with a lower synergy. There were 13 cell lines harboring *SRSF2* mutation with a lower synergy as opposed to 8 cell lines with a higher synergy.

## 3. Discussion

We assessed the effect of the most widely used first-line AML drugs, cytarabine and daunorubicin, on transcription profiles. This led to the identification of EGFR and MEK inhibitors as the most suitable combination partners for daunorubicin. These combinations had a synergistic effect when combined with daunorubicin, with minimal toxicity when deployed alone. These findings underscore the importance of transcriptional response in drug-mediated cytotoxicity and their utility in formulating better drug combinations. Further preclinical investigations are needed to compare personalized gene expression changes-based combinations with standard combinations which could expand the treatment options for older AML patients that are not eligible for intensive therapy or for relapsed cases.

Cytarabine and daunorubicin, the most widely used AML drugs, interfere with DNA synthesis in proliferating cells. Cytarabine, like deoxycytosine, gets incorporated in DNA and causes single-strand breaks during the S phase. Uncorrected single-strand breaks are converted to double-strand breaks leading to cell death. Daunorubicin inhibits the topoisomerase II enzyme leading to double-strand breaks, which also cause cell death. Mechanistically, gene repressions would be expected to reflect direct DNA damage at the pertinent gene loci.

In contrast, we observed the predominance of gene inductions in response to cytarabine and daunorubicin treatments. The transcriptional response to DNA damage is highly specific to the type of drug and to the resulting nature of the DNA damage. In the case of UV irradiation and bulky adducts, local and global transcriptional reduction is observed because of RNA polymerase II suppression via post-translational modifications and proteasomal degradation [16,17]. On the other hand, transcriptional suppression after anthracycline treatments is limited to the site of actual damage [18,19]. This is also consistent with our previous observation that the transcriptional response of AML cells to etoposide, a topoisomerase II poison, treatment also exhibited predominant gene induction [13]. Hence, most gene expression changes we observed after treatment with AML standard drugs were likely secondary, warranting further investigation to identify those contributing to cytotoxicity.

Pathway analysis revealed the activation of treatment-relevant processes, including chemotaxis, leukocyte migration, differentiation, and migration. Along with its cytotoxic effect, cytarabine has been known to induce AML cell differentiation at lower doses [20]. We also identified certain pathways which were treatment-specific. Cytarabine and the combination treatment resulted in the activation of ERK signaling and pyrimidine metabolism. IL-6 production was activated in response to daunorubicin and the combination treatment.

Interestingly IL-6 was not associated with HL-60 cells which exhibited poor response to daunorubicin treatment. However, these cells showed IL-6 activation after the combination treatment. This indicates the involvement of proinflammatory cytokines in response to standard chemotherapeutics. The standard “7 + 3” AML treatment activates pro-inflammatory cytokines [21]. NF-kB signaling activates pro-inflammatory cytokines, including IL-6 [22,23]. A recent study demonstrated activation of NF-kB signaling in 50% of AML patients 48 h after the standard treatment [24]. The absence of an inflammatory response in the case of HL60 cells could indicate an inadequate treatment response. This is most likely due to the deletion of *TP53* in HL60 cells, a major regulator of the DNA damage response [25]. Loss of *TP53* can sustain cell growth via activated RAF/MEK/ERK signaling [26]. This is evident from activating the PI3K pathway only in HL60 cells after daunorubicin treatment. Phosphorylation and subsequent activation of PI3K have been associated with resistance to anthracyclines, including daunorubicin [27]. This may have contributed to the daunorubicin resistance in HL60, which was overridden by the combination treatment with EGFR and MEK inhibitors.

Instead of targeting the aforementioned processes individually, we derived a comprehensive profile of gene expression changes depicting either response or the lack of response to daunorubicin in AML cells. By comparing gene expression changes after individual treatments and their combination across all three AML cell lines, we identified potential daunorubicin combination partners. Experimental validation confirmed the synergistic response of EGFR and MEK inhibitors. EGFR, a tyrosine kinase, is known to be involved in various cancers, including breast and non-small cell lung cancer, because of its proliferative and anti-apoptotic activities [28]. EGFR is rarely mutated in AML patients. However, its expression is associated with poor AML prognosis [29,30]. Clinical trials show limited applicability of EGFR inhibitors as standalone agents in AML patients [31,32]. Our data indicates that EGFR inhibitors are ideal candidates to be combined with daunorubicin, most likely via inhibition of daunorubicin-activated PI3K signaling.

MAP-ERK kinases (MEKs) are serine/threonine kinases that respond to stress signaling and are involved in cell survival and apoptosis [33]. MEKs are frequently dysregulated in AML and myelodysplastic syndrome (MDS), making them attractive targets for therapeutic interventions [34]. Previous reports have shown that MEK inhibitors sensitize cancer cells by inhibiting ERK1/2, which is usually activated by daunorubicin-mediated phosphorylation [35,36,37]. We observed a synergistic response of HL60 cells to the combination of PD98059, a MEK inhibitor specifically inhibiting MEK1-mediated activation of MAPK, and daunorubicin. Interestingly, PD98059 exhibited very little standalone toxicity, indicating that the synergistic response is most likely via modulating opposing biological processes. MEK inhibitors are also being explored in various clinical trials as standalone agents (clinical trial NCT02089230) and combinations (clinical trialös NCT02105350 and NCT02041481). Clinically, MEK inhibitors are considered non-effective as a standalone agent [38,39,40]. This is consistent with our observation that MEK inhibitor PD98059 is not cytotoxic alone, even at high dosages. MEK inhibition as a combination strategy in addition to the standard “7 + 3” (clinical trial NCT02049801) or azacitidine and venetoclax (clinical trial NCT04487106) is currently being investigated for relapsed or refractory AML cases in several clinical trials [41].

Currently, gene mutations play a critical role in guiding the choice of AML drugs [6,42]. Our gene expression-based combinations of daunorubicin and EGFR inhibitors did not correlate with the most common AML mutations, including *FLT3*, *IDH2*, and *NRAS*. This indicates that the synergistic effects of the daunorubicin and EGFR inhibitors could be applicable to a wide number of AML samples. In most cases, the targeted drugs are insufficient as a single agent and need to be combined with existing classical drugs [6]. Hence, the effectiveness of EGFR inhibitors as “add-ons” to the targeted therapy supplemented with daunorubicin needs to be evaluated. The broader activity of daunorubicin-EGFR inhibitor combinations is also evident from the pan-cancer analysis. In this case, we found *IGF1R* and *PRKCD* mutations associated with increased sensitivity to the combination. Insulin-like growth factor 1 receptor (IGF1R) is a tyrosine kinase associated with cell growth and survival. It is also involved in the DNA damage tolerance pathway by rescuing DNA damage-stalled replication fork [43]. Protein kinase C delta (PRKCD) is a serine/threonine-protein kinase involved in several processes, including DNA damage response and apoptosis, and is also associated with EGFR signaling [44,45]. The enhanced synergy of cancer cells harboring *PRKDC* mutation to the daunorubicin-EGFR inhibitor combination could result from inhibiting nonhomologous end joining-mediated DNA double-strand breaks repair [44]. To summarize, we show that the response of AML cells to the standard chemotherapeutic drugs comprises more than DNA double-strand break-mediated gene repression. Most transcriptional changes seem to be secondary to the double-strand breaks. These transcriptional changes play a vital role in understanding the response, or non-response, to the standard AML drugs. Importantly, transcriptional changes allow the identification of synergistic combinations which could be widely applicable due to their limited standalone toxicity.

An apparent limitation of this work is the use of a small cohort of AML cell lines. However, this approach better represents a clinical scenario where individual patients need to be evaluated for the best possible drug treatment. Crucially, the combinations identified in our study were experimentally confirmed as synergistic. Furthermore, the analysis of previously published drug combination screens confirmed the synergistic effect of the combinations beyond the cell lines in which they were predicted [46,47]. Such transcriptional response-based combinations could be of huge advantage, especially for patients that can’t tolerate intensive chemotherapy and for refractory cases that don’t respond to standard combination therapy. To refine the treatment of such aggressive AML cases, further preclinical investigations using ex vivo samples and in vivo, mouse models are required to capture heterogeneity in treatment-evoked gene expression changes. The described approach applies to other cancer drugs and types of cancers, including solid tumors. The major limitation of this approach is the unavailability of gene expression changes before initiating the treatment. This can be addressed by using pre-existing non-responding samples for discovering drug combinations. As seen from our data, gene expression change-based drug combinations are applicable beyond the samples used for their discovery. Furthermore, we believe that further computational advances will facilitate the prediction of the transcriptional response to retrospectively formulate personalized drug combinations, bypassing the need to monitor such gene expression changes in patients. We envision personalised drug combinations based on the predicted gene expression changes to improve significantly current chemotherapy regimens.

## 4. Materials and Methods

### 4.1. Cell Culture and Drugs Treatment

Acute Myeloid Leukemia (AML) cell lines HL-60, MOLM-13, and OCI-AML3 were purchased from Deutsche Sammlung von Mikroorganismen und Zellkulturen (DSMZ, Braunschweig, Germany). Cell lines were maintained at 37 °C and 5% CO_2_ in appropriate media (HL60 and MOLM13 in RPMI medium (Sigma-Aldrich, Darmstadt, Germany) containing 10% heat-inactivated FBS (Biochrom, Berlin, Germany) and OCIAML3 in alpha-MEM medium (Sigma-Aldrich, Darmstadt, Germany) containing 20% heat-inactivated FBS). Cell lines were routinely verified for mycoplasma contamination using Venor^®^GeM Mycoplasma Detection Kit (Sigma-Aldrich, Darmstadt, Germany). Cell lines were authenticated by Multiplexion, Germany. The inhibitors cytarabine, daunorubicin, and vorinostat were purchased from Selleckchem (Houston, TX, USA). The MEK inhibitor PD98059 was purchased from Merck, Darmstadt, Germany.

### 4.2. Primary Patient Samples

Heparin-treated bone marrow samples were obtained from patients with AML treated at the University Medical Center (Mainz, Germany). Informed consent was obtained in accordance with the Declaration of Helsinki, and all the laboratory experiments using primary samples were approved by the local ethics committee. Mononuclear cells were maintained for 48 h in Iscove’s modified Dulbecco’s media containing BIT (15%), beta-mercaptoethanol (20 µM), stem cell factor (100 ng/mL), FLT3-L (50 ng/mL), interlukin 3 (20 ng/mL), GM-CSF (20 ng/mL), thrombopoietin (50 ng/mL), stemregenin 1 (0.75 µM), UM171 (35 nM), and penicillin-streptomycin (1%) [48]. Afterwards, the cells were treated with drugs for 24 h in Iscove’s modified Dulbecco’s media containing 20% FBS.

### 4.3. CellTiter-Glo Cell Toxicity Assay

Cell viability in response to drug treatments was measured using CellTiter Glo^®^ 2.0 assay (Promega, Walldorf, Germany) according to the manufacturer’s instructions. In short, 5000 cells were seeded per well in a 96-well plate containing 100 µL media and treated with cytarabine (0.1 µM) and daunorubicin (0.02 µM) alone and in combination for 24 h. After the treatment, 100 µL Glo reagent was added to each well, and chemiluminescence was measured after 10 min incubation in the dark at room temperature. The percentage of cell death was calculated by comparing the luminescence of drug-treated cells with the DMSO-treated cells (considered 100% viable).

### 4.4. RNA Extraction and Library Preparation

The effect of drugs cytarabine and daunorubicin on AML cell transcription profiles was determined by RNA-Sequencing as described before [13]. In short, 1 × 10⁶ cells were seeded in a 6-well plate containing 5 mL media and treated with cytarabine (0.1 µM) and daunorubicin (0.02 µM) alone and in combination for 24 h. Total RNA was then isolated using Trizol (Thermo Fisher Scientific, Braunschweig, Germany), Monarch Total RNA Miniprep kit (New England Biolabs, Frankfurt, Germany), and DNase I Digest kit (VWR PEQLAB GmbH, Erlangen, Germany) according to manufacturer’s instructions. Samples with RIN ≥ 5.8 were sequenced on a NovaSeq PE150 platform at the Novogene Company Limited (Cambridge, UK) at a depth of 30 million reads.

### 4.5. RNA-Seq and Downstream Analysis

The quality of raw sequencing reads was assessed using FastQC version 0.11.9 (Babraham Bioinformatics, Cambridge, UK) [49]. The transcripts from the RNA-Seq reads were quantified using the decoy-aware selective alignment mode of the Salmon (version 1.8.0) [50]. The reference transcriptome and genome assemblies (GRCh38) were obtained from Ensembl. The entire genome sequence was used as the decoy sequence by concatenating this genome at the end of the transcript sequences (coding and non-coding) and populating the decoy file with chromosome names. The quantified transcript counts were summed to gene counts using the tximeta package (version 1.14.0) in R [51,52]. These counts were used to identify differentially expressed genes between drug- and DMSO-treated cells using R’s edgeR package (version 3.38.1) [53]. Genes exhibiting log2 fold change greater than |1| with a false discovery rate below 0.05 were considered significant for downstream analyses. RNA-Seq counts and raw sequencing reads are available at GEO accession number GSE227269 [54]. The list of genes essential for AML cell survival was obtained from the CRISPR-Cas9 screen from the Cancer Dependency Map (DepMap) portal (Broad Institute, Cambridge, MA, USA) [14,15]. The pathways associated with gene expression changes were identified using the clusterProfiler package in R (version 4.4.4) [55]. Functional enrichment analysis was performed using g:Profiler (version e108_eg55_p17) using the “multiquery” option [56]. All associations with *p*-adjusted values below 0.05 were considered significant.

### 4.6. Daunorubicin Combination Identification

To identify transcription profile-based drug combinations, gene expression changes after monotherapy and combination therapy were compared across 3 AML cell lines. We reasoned that by comparing treatment-evoked gene expression changes in responsive cells with non-responsive cells, we could enrich sensitivity-defining genes that could be targeted to enhance the cytotoxic response. The daunorubicin-resistant (DNRResist) profile was derived from HL60 cells using gene expression changes after only daunorubicin treatment which was absent in MOLM13 and OCIAML3 cells after both daunorubicin and the combination treatments. Analogously, the daunorubicin-response (DNRResponse) profile was constructed using gene expression changes common to MOLM13 and OCIAML3 cells which were absent in HL60 cells after both daunorubicin and the combination treatments. The DNRResist and DNRResponse profiles were submitted to the online CLUE platform to identify other drugs and genetic modulations with similar transcriptional responses [57]. Normalized connectivity score was used to identify perturbations with either similar (score > 0) or opposite (score < 0) transcriptional response. The overlapping perturbations between an opposite transcriptional response to the DRNResist profile and a similar transcriptional response to the DNRResponse profile were further filtered to remove ambiguous discoveries (e.g., perturbations with similar effect after knockdown as well as overexpression and perturbations with a similar effect as both DNRResist and DNRResponse profiles). To further enrich the true positives, inhibitors with similar transcriptional effects as their target gene counterparts were selected. The potential daunorubicin combination partners were then ranked based on the observed transcriptional effect in the number of cell lines.

### 4.7. Daunorubicin Combination Verification

To validate the predicted combination partners, the viability of HL60 cells was measured in response to the combinations of PD98059 (MEK inhibitor) and vorinostat (HDAC inhibitor) with daunorubicin using a 5 × 3 concentration matrix. Similarly, the viability of MOLM13, OCIAML3, and a primary AML sample was measured in response to the combination of PD98059 and daunorubicin. The cell viability was calculated using the CellTitre Glo assay described above (section CellTiter-Glo cell toxicity assay). The effect of the combination was evaluated using the Bliss independence test of the SynFinder portal [58]. The combination with the BLISS score above 0 was considered synergistic, while a negative BLISS score was considered an antagonistic effect. To validate other predicted combination partners and evaluate the broader applicability of these combinations, a large drug combination screen NCI ALMANAC was accessed using the database DrugComb [46,47]. The average BLISS score was calculated for each class of the inhibitor to identify synergistic combinations.

### 4.8. Statistical Association of the Drug Synergy with Genomic Markers

To investigate whether the mutational background of cancer cell lines influences the synergistic response of gene expression-based drug combinations, we correlated genomic markers by fitting an ANOVA model [59,60]. We obtained the genome sequencing-based gene mutation data for 56 pan-cancer cell lines from the Cell Model Passports portal [61]. We utilized average BLISS synergy scores of EGFR inhibitors in combination with daunorubicin for pan-cancer analysis. An ANOVA model was fitted to calculate the effect size for each gene-drug association, and the resulting *p*-values were corrected using the Tibshirani-Storey method implemented in the GDSCTools python package (version 1.0.1) [62]. The default threshold for *p*-value (0.001) and FDR (25%) was used to identify significant associations. The ten most frequently mutated genes in AML patients were obtained from cBioPortal [63,64]. The volcano plot was constructed using the EnhancedVolcano package (version 1.14.0) in R [65]. Based on the BLISS synergy scores, the top 50% of cell lines were referred to as those with higher synergy and the bottom 50% as those with lower synergy.

## Data Availability

The RNA sequencing data included in this work have been deposited in NCBI’s Gene Expression Omnibus (GEO) database with the accession number GSE227269 (https://www.ncbi.nlm.nih.gov/geo/query/acc.cgi?acc=GSE227269, accessed on 13 March 2023).

## Supplementary Materials

The following supporting information can be downloaded at: https://www.mdpi.com/article/10.3390/ijms241612926/s1.
