# Supplementary material for: Transcriptional Response to Standard AML Drugs Identifies Synergistic Combinations
Source: Int J Mol Sci. 2023 Aug 18;24(16):12926. doi: 10.3390/ijms241612926 (PMC10455220; doi:10.3390/ijms241612926)
Supplement: Supplementary file 1 [file ijms-24-12926-s001.zip › Supplementary Figures.pdf]

Transcriptional Response to Standard AML Drugs Identifies Synergistic Combinations

Piyush More [1,*], Joëlle Aurelie Mekontso Ngaffo [1,2], Ute Goedtel-Armbrust [1], Patricia S. Hähnel [3,4], Udo F. Hartwig [4,5], Thomas Kindler [3,4], Leszek Wojnowski [1]

[1] Department of Pharmacology, University Medical Center, Johannes Gutenberg-University, 55131 Mainz, Germany

[2] Leibniz Institute for New Materials, 66123 Saarbrücken, Germany

[3] University Cancer Center (UCT) Mainz, Johannes Gutenberg-University, 55131 Mainz, Germany

[4] Department of Hematology & Medical Oncology, University Medical Center, Johannes Gutenberg-University, 55131 Mainz, Germany

[5] Research Center of Immunotherapy, University Medical Center, Johannes Gutenberg-University, 55131 Mainz, Germany

*Author to whom the correspondence should be addressed, piyusmor@uni-mainz.de

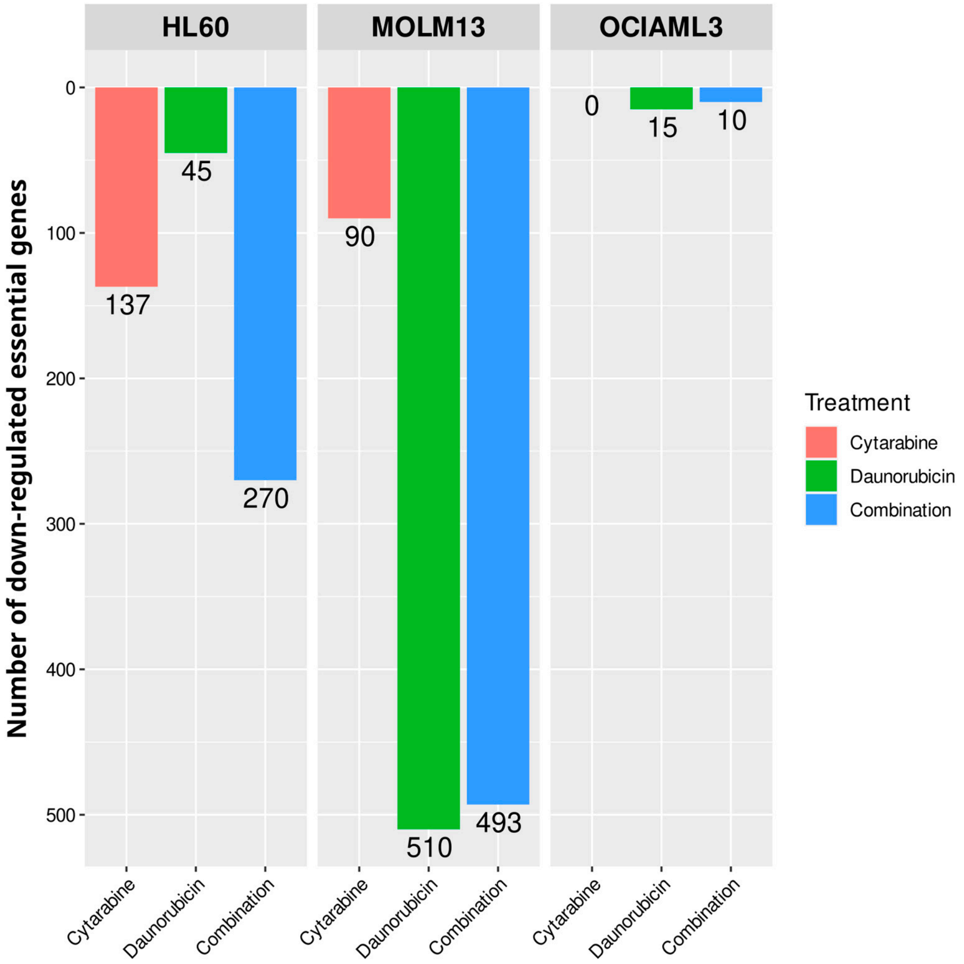

Supplementary Figure S1. Down-regulated essential genes. The number of essential genes down-regulated after 24 hours treatment with cytarabine, daunorubicin, and their combination. The list of essential genes was obtained from the Cancer Dependency Map (DepMap) portal (Broad Institute, US).

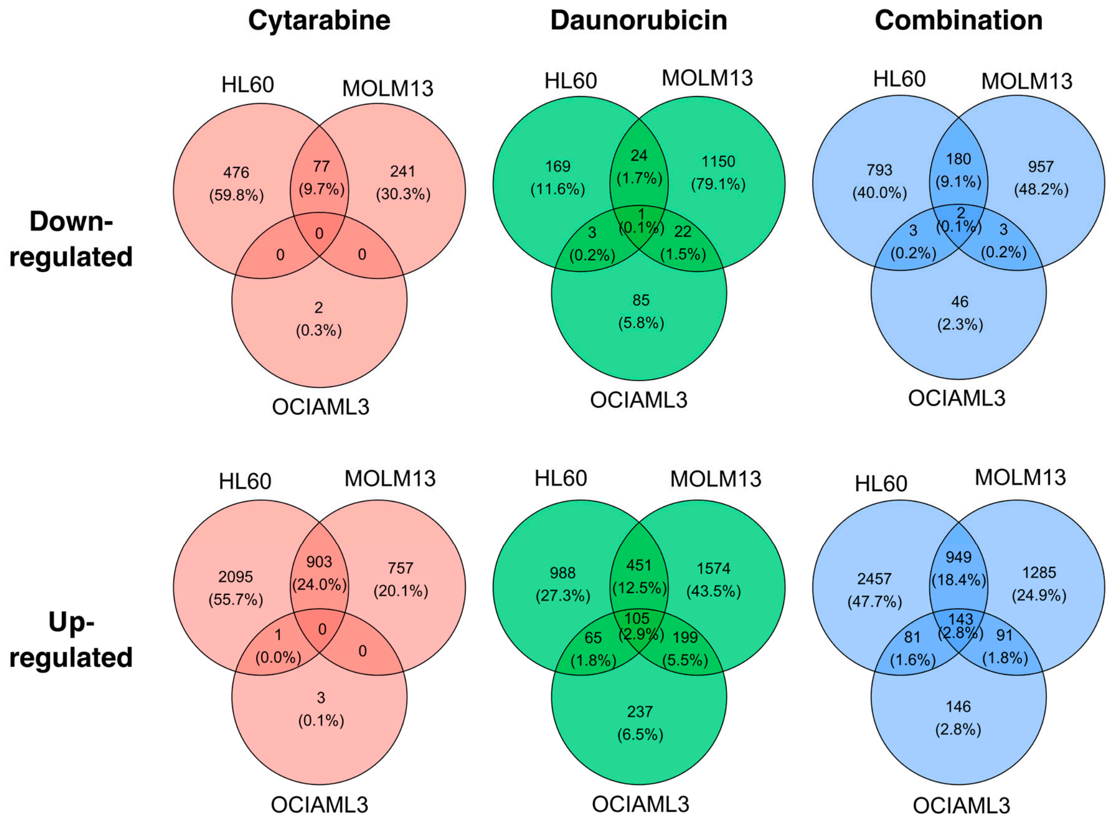

Supplementary Figure S2. Treatment-wise overlapping gene expression changes. The venn diagrams represent overlap between gene expression changes that occurred after treatment with cytarabine, daunorubicin, and the combination for 24 hours across all AML cell lines.

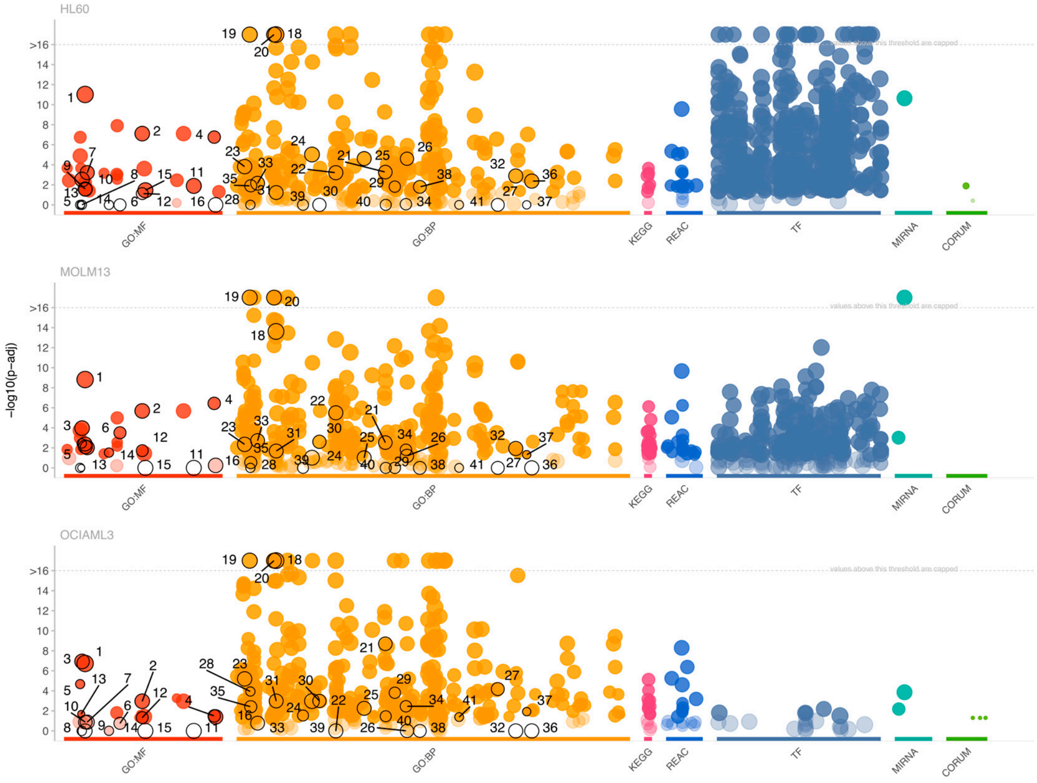

id	source	term_id	term_name	term_size	p_value HL60	p_value MOLM13	p_value OCIAML3
1	GO:MF	GO:0005515	protein binding	14778	9.5e−12	1.5e−09	1.8e−07
2	GO:MF	GO:0038023	signaling receptor activity	1489	7.7e−08	2.1e−06	1.1e−03
3	GO:MF	GO:0005102	signaling receptor binding	1556	3.0e−03	1.1e−04	1.2e−07
4	GO:MF	GO:0140375	immune receptor activity	142	1.7e−07	3.6e−07	3.2e−02
5	GO:MF	GO:0004875	complement receptor activity	11	1.0e+00	1.0e+00	2.1e−05
6	GO:MF	GO:0030527	structural constituent of chromatin	101	1.0e+00	3.1e−04	1.7e−01
7	GO:MF	GO:0008289	lipid binding	825	6.1e−04	9.5e−03	1.3e−01
8	GO:MF	GO:0005035	death receptor activity	13	1.0e+00	3.6e−03	1.0e+00
9	GO:MF	GO:0005509	calcium ion binding	724	2.6e−02	4.1e−03	1.0e+00
10	GO:MF	GO:0005543	phospholipid binding	471	2.9e−02	8.0e−03	1.3e−01
11	GO:MF	GO:0097367	carbohydrate derivative binding	2300	1.3e−02	1.0e+00	1.0e+00
12	GO:MF	GO:0038024	cargo receptor activity	85	7.5e−02	1.9e−02	4.7e−02
13	GO:MF	GO:0004982	N−formyl peptide receptor activity	4	1.0e+00	1.0e+00	2.0e−02
14	GO:MF	GO:0017154	semaphorin receptor activity	12	1.0e+00	3.0e−02	9.7e−01
15	GO:MF	GO:0043168	anion binding	2426	3.5e−02	1.0e+00	1.0e+00
16	GO:MF	GO:0140677	molecular function activator activity	1284	1.0e+00	5.6e−01	4.0e−02
18	GO:BP	GO:0007154	cell communication	6573	1.2e−28	2.4e−14	1.8e−20
19	GO:BP	GO:0002376	immune system process	2683	2.6e−17	3.2e−22	1.2e−26
20	GO:BP	GO:0006952	defense response	1791	2.1e−18	8.1e−23	1.0e−21
21	GO:BP	GO:0042060	wound healing	440	5.4e−04	3.0e−03	2.0e−09
22	GO:BP	GO:0030029	actin filament−based process	813	6.2e−04	3.2e−06	1.0e+00
23	GO:BP	GO:0001932	regulation of protein phosphorylation	1101	1.5e−04	4.5e−03	6.4e−06
24	GO:BP	GO:0016310	phosphorylation	1872	9.4e−06	9.7e−02	1.0e−03
25	GO:BP	GO:0034330	cell junction organization	708	2.4e−05	9.9e−02	5.6e−03
26	GO:BP	GO:0045229	external encapsulating structure organization	324	2.5e−05	6.3e−02	1.0e+00
27	GO:BP	GO:0072593	reactive oxygen species metabolic process	225	1.0e+00	1.0e+00	6.6e−05
28	GO:BP	GO:0002430	complement receptor mediated signaling pathway	12	1.0e+00	1.0e+00	1.2e−04
29	GO:BP	GO:0043277	apoptotic cell clearance	52	1.5e−02	1.0e+00	1.5e−04
30	GO:BP	GO:0019058	viral life cycle	316	1.0e+00	2.5e−03	1.0e−03
31	GO:BP	GO:0007169	transmembrane receptor protein tyrosine kinase signaling pathway	626	5.9e−02	2.1e−02	1.0e−03
32	GO:BP	GO:0097435	supramolecular fiber organization	814	1.3e−03	1.2e−02	1.0e+00
33	GO:BP	GO:0003013	circulatory system process	596	6.9e−03	1.9e−03	1.7e−01
34	GO:BP	GO:0045071	negative regulation of viral genome replication	56	8.7e−01	1.5e−02	3.5e−03
35	GO:BP	GO:0002456	T cell mediated immunity	108	1.3e−02	2.9e−01	3.9e−03
36	GO:BP	GO:0140352	export from cell	871	4.3e−03	1.0e+00	1.0e+00
37	GO:BP	GO:0110089	regulation of hippocampal neuron apoptotic process	7	1.0e+00	4.9e−02	1.2e−02
38	GO:BP	GO:0046578	regulation of Ras protein signal transduction	201	1.6e−02	1.0e+00	1.0e+00
39	GO:BP	GO:0014002	astrocyte development	40	1.0e+00	1.0e+00	2.9e−02
40	GO:BP	GO:0042119	neutrophil activation	41	1.0e+00	1.0e+00	3.4e−02
41	GO:BP	GO:0060700	regulation of ribonuclease activity	9	1.0e+00	1.0e+00	4.2e−02

g:Profiler (biit.cs.ut.ee/gprofiler)

Supplementary Figure S3. Enrichment analysis for the daunorubicin treatment. Functional gene-set enrichment analysis performed using g:Profiler for gene ontology molecular function (GO:MF), biological processes (BP), KEGG and Reactome pathways, putative transcription factor binding sites using TRANSFAC (TF), miRNA targets, and protein complex annotation using CORUM. The highlighted and labeled circles represent significant associations. The plot has been generated using a custom function from the gprofiler2 package in R (version 0.2.2).

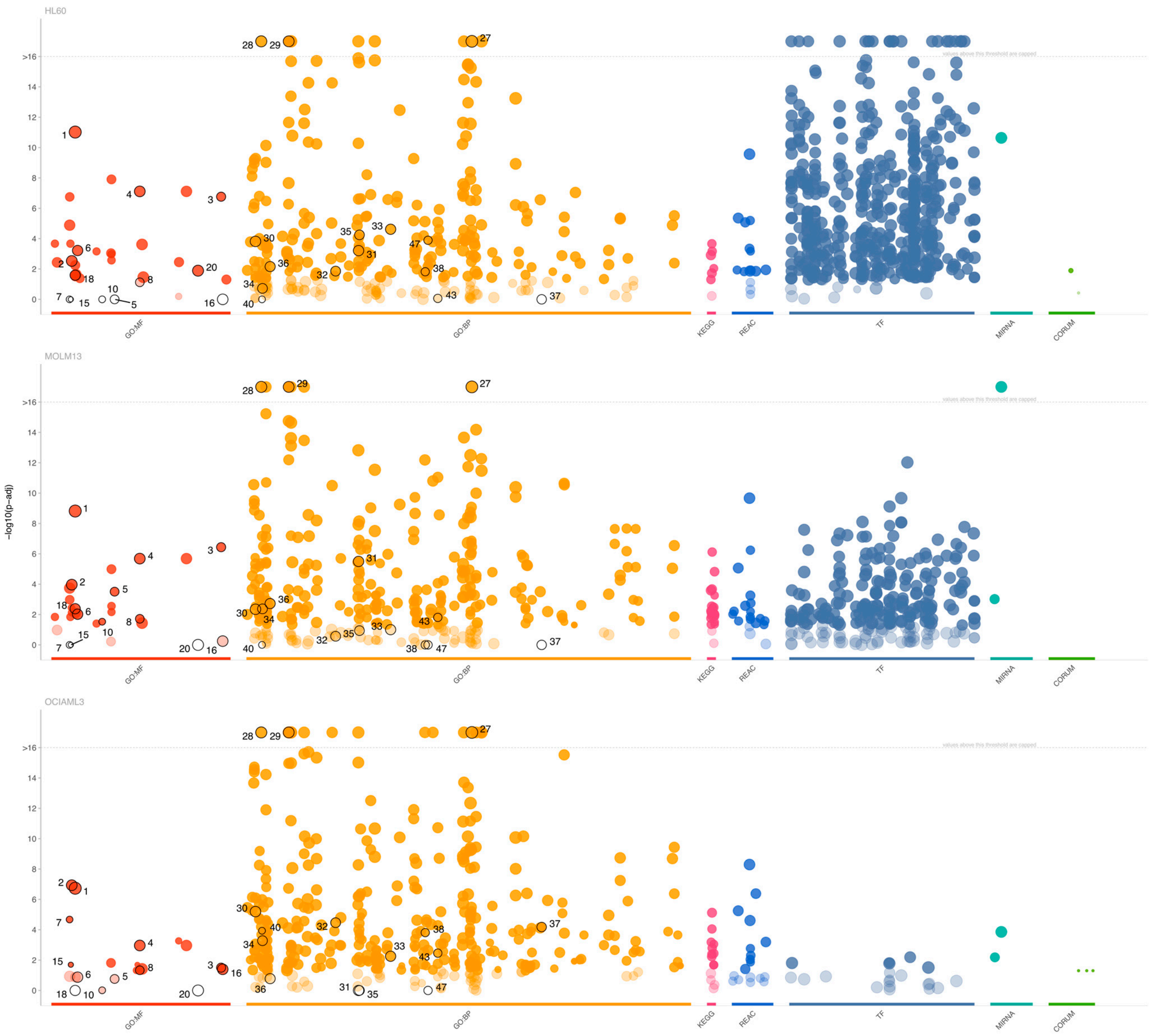

id	source	term_id	term_name	term_size		p_value HL60		p_value MOLM13		p_value OCIAML3
1	GO:MF	GO:0005515	protein binding	14778		9.5e−12		1.5e−09		1.8e−07
2	GO:MF	GO:0005102	signaling receptor binding	1556		3.0e−03		1.1e−04		1.2e−07
3	GO:MF	GO:0140375	immune receptor activity	142		1.7e−07		3.6e−07		3.2e−02
4	GO:MF	GO:0038023	signaling receptor activity	1489		7.7e−08		2.1e−06		1.1e−03
5	GO:MF	GO:0030527	structural constituent of chromatin	101		1.0e+00		3.1e−04		1.7e−01
6	GO:MF	GO:0008289	lipid binding	825		6.1e−04		9.5e−03		1.3e−01
7	GO:MF	GO:0004875	complement receptor activity	11		1.0e+00		1.0e+00		2.1e−05
8	GO:MF	GO:0038024	cargo receptor activity	85		7.5e−02		1.9e−02		4.7e−02
10	GO:MF	GO:0017154	semaphorin receptor activity	12		1.0e+00		3.0e−02		9.7e−01
15	GO:MF	GO:0004982	N−formyl peptide receptor activity	4		1.0e+00		1.0e+00		2.0e−02
16	GO:MF	GO:0140677	molecular function activator activity	1284		1.0e+00		5.6e−01		4.0e−02
18	GO:MF	GO:0005509	calcium ion binding	724		2.6e−02		4.1e−03		1.0e+00
20	GO:MF	GO:0097367	carbohydrate derivative binding	2300		1.3e−02		1.0e+00		1.0e+00
27	GO:BP	GO:0050896	response to stimulus	9039		1.3e−22		3.1e−21		4.2e−26
28	GO:BP	GO:0002376	immune system process	2683		2.6e−17		3.2e−22		1.2e−26
29	GO:BP	GO:0006952	defense response	1791		2.1e−18		8.1e−23		1.0e−21
30	GO:BP	GO:0001932	regulation of protein phosphorylation	1101		1.5e−04		4.5e−03		6.4e−06
31	GO:BP	GO:0030029	actin filament−based process	813		6.2e−04		3.2e−06		1.0e+00
32	GO:BP	GO:0018212	peptidyl−tyrosine modification	381		1.4e−02		2.7e−01		3.4e−05
33	GO:BP	GO:0034330	cell junction organization	708		2.4e−05		9.9e−02		5.6e−03
34	GO:BP	GO:0002460	adaptive immune response based on somatic recombination of immune receptors built from immunoglobulin superfamily domains	355		1.9e−01		4.2e−03		5.2e−04
35	GO:BP	GO:0030198	extracellular matrix organization	321		5.7e−05		1.1e−01		1.0e+00
36	GO:BP	GO:0003013	circulatory system process	596		6.9e−03		1.9e−03		1.7e−01
37	GO:BP	GO:0072593	reactive oxygen species metabolic process	225		1.0e+00		1.0e+00		6.6e−05
38	GO:BP	GO:0043277	apoptotic cell clearance	52		1.5e−02		1.0e+00		1.5e−04
40	GO:BP	GO:0002430	complement receptor mediated signaling pathway	12		1.0e+00		1.0e+00		1.2e−04
43	GO:BP	GO:0045071	negative regulation of viral genome replication	56		8.7e−01		1.5e−02		3.5e−03
47	GO:BP	GO:0043551	regulation of phosphatidylinositol 3−kinase activity	56		1.3e−04		1.0e+00		1.0e+00

g:Profiler (biit.cs.ut.ee/gprofiler)

Supplementary Figure S4. Enrichment analysis for the combination treatment. Functional gene-set enrichment analysis performed using g:Profiler for gene ontology molecular function (GO:MF), biological processes (BP), KEGG and Reactome pathways, putative transcription factor binding sites using TRANSFAC (TF), miRNA targets, and protein complex annotation using CORUM. The highlighted and labeled circles represent significant associations. The plot has been generated using a custom function from the gprofiler2 package in R (version 0.2.2).

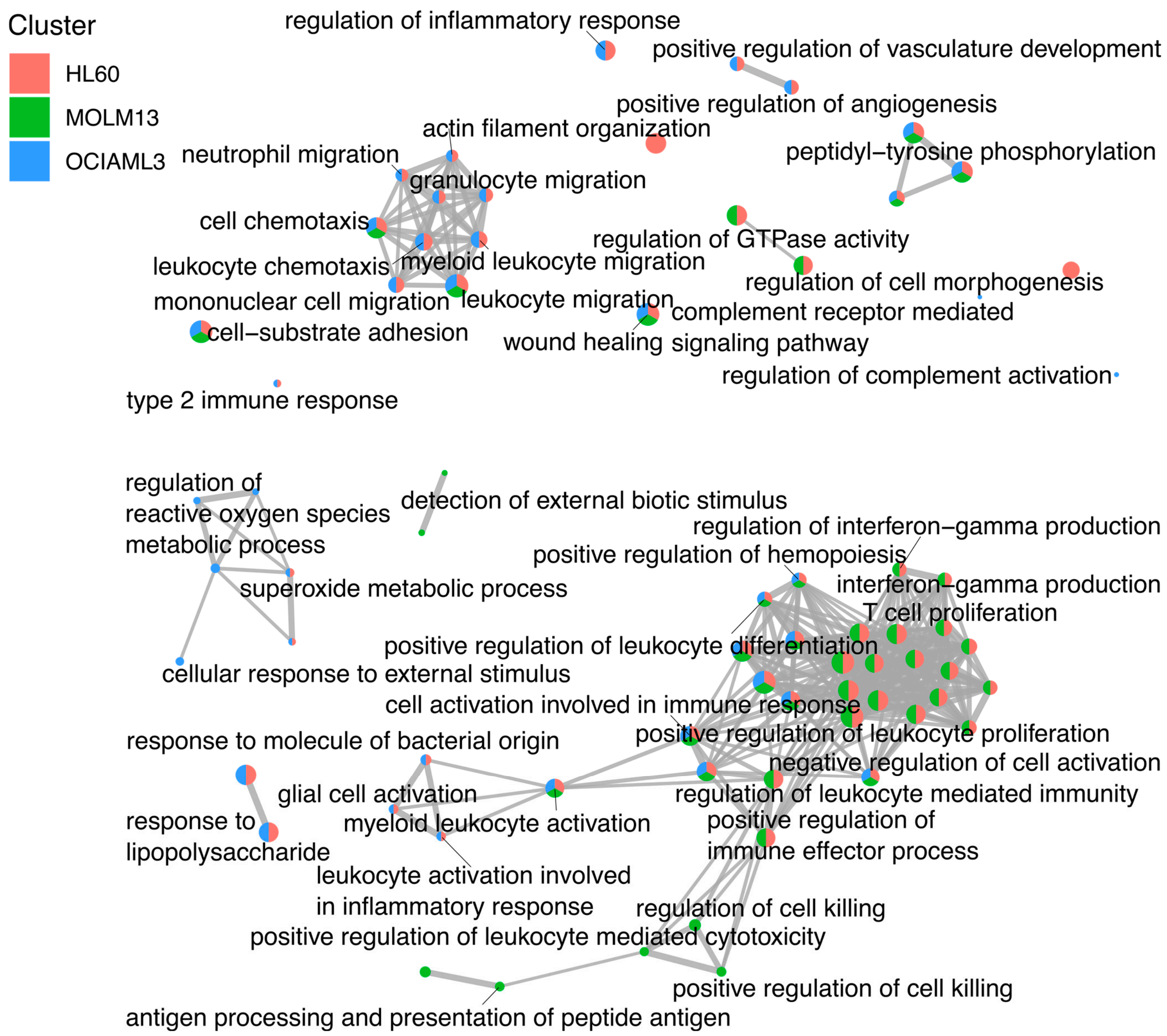

Supplementary Figure S5. Gene network map after combination treatment. A network map organizing enriched biological processes into a network with edges connecting overlapping gene sets. The network clustered mutually overlapping gene sets together. The clusters represent enriched biological processes after the combination treatment (cytarabine + daunorubicin) in 3 AML cell lines.

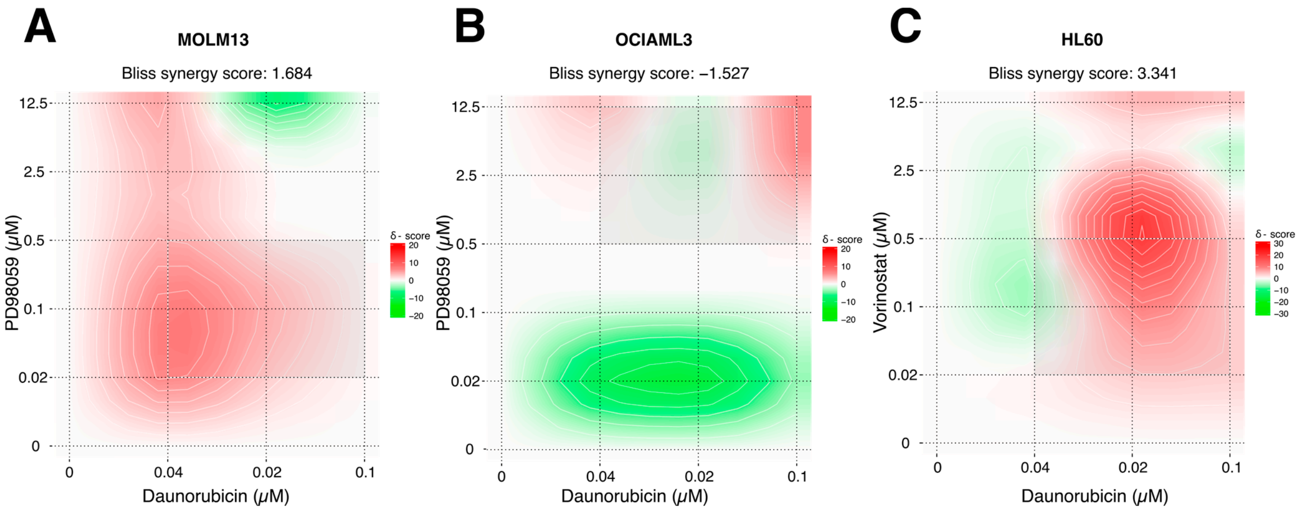

Supplementary Figure S6. The effect of daunorubicin combinations in AML cell lines. The contour plots representing the effect of daunorubicin combined with PD98059 in (A) MOLM13, (B) OCIAML3, and (C) the effect of daunorubicin combined with vorinostat in HL60 cells.

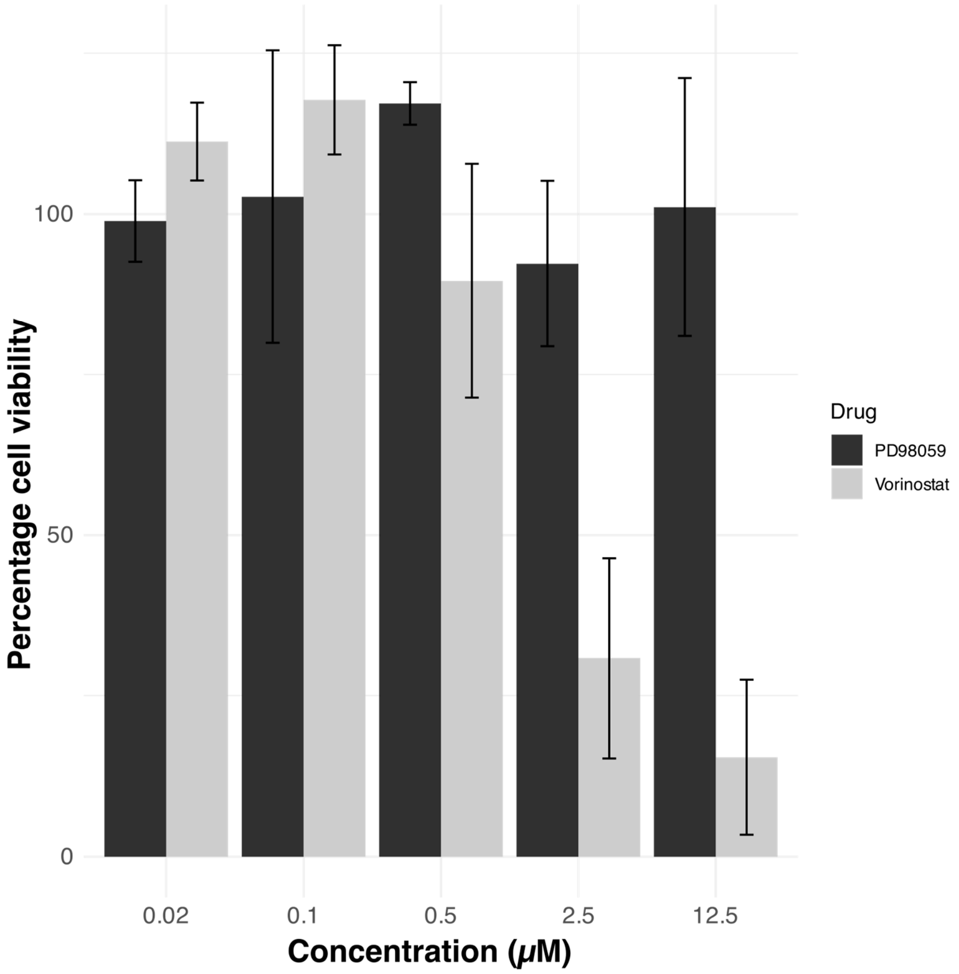

Supplementary Figure S7. Standalone toxicity of MEK and HDAC inhibitors. CellTiter-Glo-based percentage cell viability of HL60 cells in response to MEK inhibitor PD98059 and HDAC inhibitor vorinostat. The viability is calculated against DMSO-treated cells.

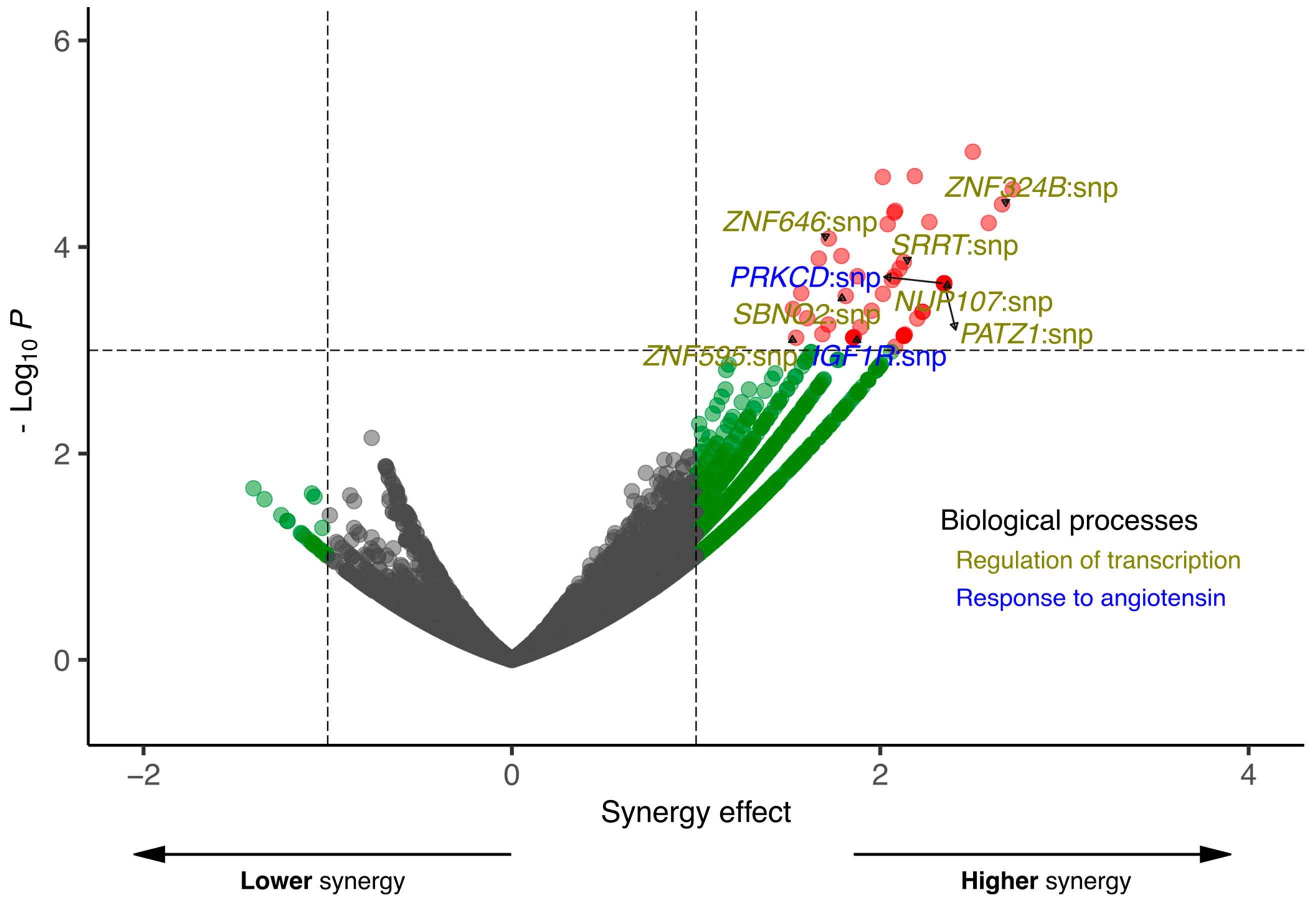

Supplementary Figure S8. Association of genetic mutations to the daunorubicin-EGFRi synergy. The volcano plot representing the effect of genetic mutations on the extent of synergy to the combination of daunorubicin and EGFR inhibitor. Genetic mutations from 56 pan-cancer cell lines and Bliss synergy scores were used to fir an ANOVA model. Labeled mutations are associated with either regulation of transcription or response to angiotensin (response to chemicals) processes.
